# Newborn Sickle Cell and Thalassaemia Screening Programme: Automating and Enhancing the System to Evaluate the Screening Programme

**DOI:** 10.3390/ijns5030030

**Published:** 2019-08-31

**Authors:** Catherine Coppinger, Robyn O’Loughlin

**Affiliations:** NHS Sickle Cell and Thalassaemia Screening Programme, Public Health England, London SE1 8UG, UK

**Keywords:** sickle cell, thalassaemia, screening, newborn, outcome data, evaluation, Agile project

## Abstract

Good information is needed to demonstrate that a screening programme is meeting its objectives, to measure performance against standards and to ensure that action is taken if standards are not met. In 2010, the NHS Sickle Cell and Thalassaemia (SCT) Screening Programme established a process to collect data on the main outcome measures for newborn babies. In 2016, a review identified that data completeness and quality relied on manual processes and there was widespread dissatisfaction amongst data providers due to duplication of data entry, poor feedback and lack of oversight of the baby to ensure safe handover from screening into treatment services. Using an Agile service design process and following the Government Digital Service Model, the SCT Screening Programme worked in close collaboration with users, wider stakeholders and system suppliers to design and build a new automated system. The new system ensures that the screening programme can fulfil its duty to evaluate the effectiveness of the programme, whilst pleasing the users and enhancing safety. User experience must be central to design and ongoing development to ensure that a new IT system is fit for purpose and adopted by users.

## 1. Introduction

The NHS Sickle Cell and Thalassaemia (SCT) Newborn Screening Programme in England aims to improve infant health through prompt identification of affected babies and timely transition into clinical care.

Since 2006, newborn screening for sickle cell disease (SCD) has been offered to parents and carers of newborn babies as part of the blood spot screening programme. Early entry into care aims to allow timely offer of penicillin prophylaxis and ensure that parents are aware of signs and symptoms of SCD. Current programme standards require parents to receive screen positive results by 28 days and for the baby to enter paediatric care by 90 days of age [1]. Beta thalassaemia major is identified as a ‘by-product’ and babies follow the same referral pathway. In 2010, an application under Section 251 was approved by the National Information Governance Board for the NHS Sickle Cell and Thalassaemia Screening Programme to collect named data without consent on all affected babies for a limited number of data items with the aim of evaluating the programme.

The register includes:

- records of babies already in the system that require follow up;
- babies identified by newborn screening as screen positive for sickle cell disease or thalassaemia;
- babies with sickle cell disease or thalassaemia presenting clinically.

Data is used to assess:

- the health of affected babies or children;
- timeliness of entry into care and start of their treatment;
- what can be learned from babies that die.

Laboratories and centres involved in the baby’s treatment used standard Excel forms to notify the project administrator based at Kings College London (KCL) at key milestones in the screening pathway. Missing data fields were common; the project administrator chased up missing submissions until records were complete.

The database moved from KCL to the National Congenital Anomalies and Rare Disorders Registration Service (NCARDRS) at Public Health England (PHE) in April 2017 when the contract with KCL expired. The agreement was for NCARDRS to ensure secure transfer of the legacy data from 2009/2010 to 31 March 2017 and ensure the safe running of this project in its current form collecting the same dataset [2]. 

An evaluation of the performance of the national SCT programme against the 2011 programme and clinical standards, published in November 2017 [3], showed that screening was accurately identifying affected babies, coverage (the number of eligible babies that are actually tested) was excellent, the test was acceptable to most parents, with approximately 2/1000 refusing the test, and that there were no known missed cases.

However, it also showed that there was scope for improvement. Delays between babies’ positive screening results and enrolment into care and acceptance of penicillin were reported, requiring optimisation of fail-safe follow-up. Only 80% of babies were enrolled into specialist care by 3 months of age and 80% of eligible babies received penicillin by 3 months of age. Additionally, penicillin adherence was highlighted as an ongoing challenge.

In 2016, a detailed review (Discovery exercise) into all aspects of the process, business interests and stakeholder requirements identified dissatisfaction with current manual processes and was the driving force behind the business case for a new IT system to automate and enhance the system for referring screen positive babies into treatment and evaluating the programme.

The business case was approved by PHE in December 2017 and in 2018, Medical Data Solutions and Systems (MDSAS) were awarded the contract following an open tender.

The SCT Newborn Outcomes System aims to

- improve patient safety by allowing users to view the status of patients along the care pathway;
- alert clinicians when important milestones (timely results to parents and entry into paediatric care) are breached;
- improve the quality and completeness of data to evaluate the programme;
- reduce duplication of data entry by integrating with the National Haemoglobinopathy Register (NHR), a database of patients with haemoglobinopathies living in the UK and NCARDRS;
- reduce manual chasing through automated prompts;
- improve reporting so that users can monitor local performance and return annual data to PHE.

The SCT Newborn Outcomes System was developed using an Agile service design process, following the Government Digital Service Model of Discovery, Alpha, Beta and Live [4].

This paper outlines the Discovery, Alpha and Beta phases of the project.

## 2. Materials and Methods

### 2.1. Discovery

The objective of the Discovery phase was to identify and prioritise the requirements of people involved in the current SCT newborn outcomes data collection process to ensure that the right solution was identified and implemented.

A number of user research sessions with the various people involved were held (Table 1). 

The interviews explored how people interacted with the current SCT newborn outcomes data collection process: what users did not like (pain points) and what they thought worked well (highlights). 

Once everyone had shared their experience, these were grouped together into categories and solutions to address the pain points were discussed and documented as user needs. Further user needs were elicited by talking through how the users wanted a future system to operate (Table 2). 

The user needs were then prioritised using the MoSCoW method (Table 3): 

The new solution would address key findings and recommendations and include failsafe activities as part of its remit. The user experience needed to be central to development to ensure user adoption.

### 2.2. Alpha Phase

The Government Digital Service manual [4] states that in the Alpha phase, you need to:

- build prototypes of your service;
- test your prototypes with users;
- demonstrate that the service you want to build is technically possible;
- find the problems with the design of your service and decide how you will solve them;
- make some estimates about how much your service will cost;
- identify the biggest risks for the Beta stage as early as possible.

Scrum methodology was used to deliver this phase of the project. The governance structure of Scrum involves a team using “Artefacts” and “Ceremonies” to manage the rapid delivery of the project. 

The team comprised:

- a Scrum Master (MDSAS Project Manager);
- a Scrum Team (MDSAS IT Business Analyst, Development, Operational and Implementation Team, PHE Project Lead, Programme Manager and Screening Technical Projects Manager);
- MDSAS and PHE Information and Clinical Governance Specialists and Clinical Safety Officers.

Artefacts and ceremonies included product backlog, sprint backlog, daily stand-up, user engagement, sprint review, showcase and retrospective.

The product backlog is the list of stories (Table 4) based on user needs identified in Discovery which are expected to complete the full project. These stories are negotiable and are subject to changes based on user involvement and ongoing development. 

The sprint backlog is the list of stories expected to be completed within a set period, called a ‘sprint’. These stories must be agreed upon before the sprint starts and completed by the end of the sprint. 

The project was broken into two-week sprints. Each sprint started with a sprint planning session and finished with a sprint review, showcase and retrospective.

At the planning session, the sprint backlog stories that were to be completed within the sprint were reviewed by the Scrum Team and the tasks to be completed were fleshed out into sub tasks, given point estimates (resource requirement) and allocated.

Daily stand-ups allowed the project team to communicate with each other about how they were moving stories to completion. 

Daily “stand-up” meetings were held by teleconference at the same time each day. Everyone from the project team reported on what they did the day before, what they would be doing that day, and any blockers stopping them from progressing their stories.

User engagement meetings were held twice per sprint to obtain vital input into the user journeys for the different user groups involved with the project. Users were recruited via professional groups Sickle Cell & Thalassaemia Association of Nurses, Midwives and Allied Professionals (STANMAP), UK Haemoglobinopathy Forum and UK Newborn Screening Laboratory Network (UKNSLN). Each of these users would actively use the system in some way. It was also important to obtain representation across the country from high and low prevalence areas and where the pathway set up varied.

At the end of each sprint, MDSAS showed a wider group of stakeholders and users what had been completed over the course of the two-week sprint.

The sprint review was attended by the entire project team and allowed the Scrum Master to close the sprint. A retrospective at the end of each sprint reviewed what went well and what could be better for each sprint. This allowed the Scrum Team to improve performance based on feedback within the team. 

Four tools were used in the delivery of this project to ensure that there was transparency between the users, PHE and the IT system supplier. 

The Jira tool was used to maintain the product backlog and sprint backlog. The tool allows stories to be grouped into epics and to assign tasks to individuals. 

Confluence was used to manage the documentation and outputs from each user story. Confluence and Jira were linked, allowing both PHE and the supplier to watch over the progress of outputs and to comment directly on any documentation. 

The Slack messaging tool was used for informal communications in an open workspace, allowing one-to-one and group or team messaging. 

Skype for Business was used for conference calling. This allowed a single phone number for

- user engagement,
- daily stand-up,
- backlog refinement.

It also allowed screens to be shared when working remotely. 

**Figure 1:**
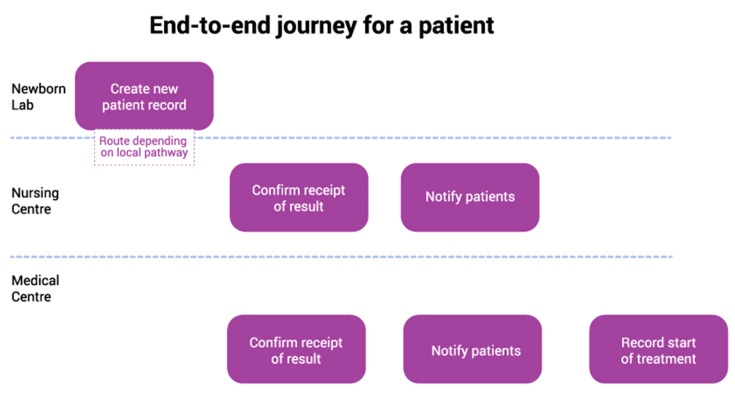
End to end user journey.

This approach allowed issues to be identified and assumptions tested early with a small user group before building software. The output for Alpha was a prototype which showed the end-to-end user journey (Figure 1) [5]. 

### 2.3. Beta Phase

The Beta phase was split into three phases: initial development of a Minimum Viable Product (MVP), Private Beta, and Public Beta. The development team continued to operate the Scrum process, with two-week sprints delivering functioning software at the end of each iteration. A small group of users supported ongoing testing and validation.

### 2.4. Initial Development

A MVP based on the user stories from Alpha was developed on the MDSAS secure N3 (NHS) server. The MVP used ‘dummy’ copies of the newborn outcome system and NHR database to avoid interfering with existing live operational software.

### 2.5. Private Beta

Once the MVP was complete, a minimum number of users were invited to use the system to complete the end to end journey across the referral pathway to replicate patient journeys in a non-live environment.

Scenarios were developed and users could access the system and follow a simple piece of data through input, onward notification, edit, report generation and export through to the end of the patient journey in the system. 

A complete journey did not mean a complete data set or user experience in the first instance. During Private Beta, the volume of information, reports and completeness of screens grew continuously. This approach involved incremental development and short feedback loops throughout the service design process. Remote user engagement workshops as well as bespoke face-to-face sessions were held weekly. Facility to feedback within the system was also available. Continuous user insight throughout the software build ensured stakeholders understood and approved the solution. A process to capture all feedback linked to the product backlog allowed the development team to review and validate bugs and change requests and make a prioritised list of the work.

This approach enabled risks to be identified and removed and gave users confidence that the solution was viable. 

### 2.6. Public Beta

In Public Beta, a functionally complete version of the software cleared of previously entered data was released. Clinical Safety Officers gave authority to release the software, having ensured that the software met the specific standards required for use in the NHS [6,7]. A small number of users were invited to use the system with live data running alongside existing processes with the understanding that this process would replace the existing process in the future.

Additional users requested to join this phase and support from PHE and MDSAS was available throughout. Training materials were developed [8] and rollout continues. 

## 3. Results

The live system provides oversight of SCT screen positive babies identified by the NHS SCT Screening Programme as they are handed over from screening to clinical services.

**Figure 2:**
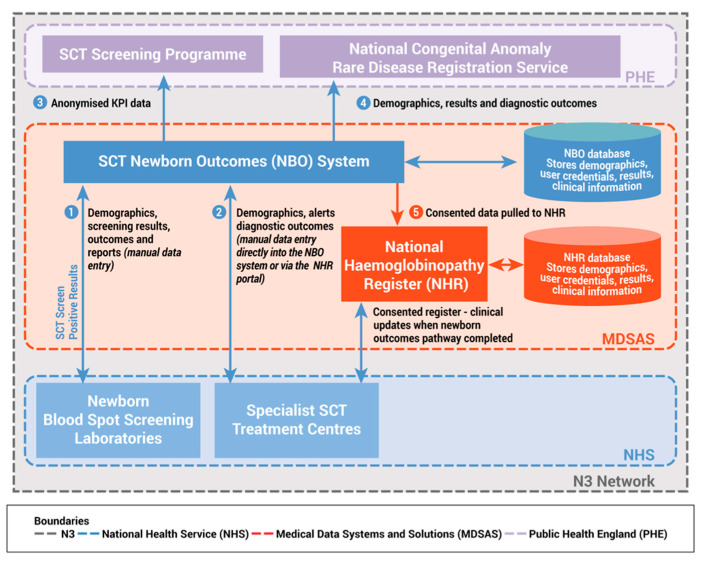
The SCT Newborn Outcomes System.

The database server is hosted in a physically secured environment (Figure 2). Secure remote access to the data is restricted to authorised user accounts. Removing the need for clinicians to enter the same data twice was one of the key requirements for the project. The referral is created in the screening laboratory (Figure 2 point 1) and shared with clinical centre(s), depending on the local pathway. There is a single record for each baby and data is added to the record by clinical teams. The SCT Newborn Outcomes System can be accessed directly or NHR users can access the system from a single login. The interface in the NHR matches the main SCT Newborn Outcomes System and is provided as a ‘view’ onto the system for NHR users. It does not affect the process or data collected; all data is held in the SCT Newborn Outcomes System (Figure 2 point 2).

De-personalised data reports on performance against standards are returned to the NHS SCT Screening Programme (Figure 2 point 3) and data is shared with NCARDRS (Figure 2 point 4), automatically replacing the current manual processes.

Clinicians in Haemoglobinopathy Centres can pull a minimum data set from the newborn outcome system into the NHR providing they confirm that parental consent has been given, diagnosis is confirmed and mandatory data fields in the outcome system are complete. No data crosses into the NHR until consent has been explicitly recorded. Data items recorded on the NHR are not shared back (Figure 2 point 5).

Users can access a comprehensive suite of reports, including a real-time data quality report of missing data items, performance against standards and results by condition, gestation and ethnicity. Users can also export their own data for specified time periods and in various formats.

Users are also notified by email when new referrals and updates to the system are made.

## 4. Discussion

User experience was central to the development of the SCT Newborn Outcome System and is essential for user adoption to be successful. This has been evident in the initial stages of implementation. The ‘pain points’ (Table 2) identified in the Discovery phase (Table 1) were systematically addressed. There is oversight of the baby as he/she negotiates a complex journey with multiple handovers from screening into treatment services.

Laboratories, nursing and medical haemoglobinopathy centres can log, track and refer patients to ensure they correctly complete the clinical pathway. Alerts are sent when key milestones are due or missed and a number of predetermined reports enable users and PHE to monitor local performance and return annual KPI data required. Duplication of data entry is reduced by linking with NCARDRS and allowing data to be pulled through to the NHR when parental consent has been given. 

Highlighting these benefits to system users was instrumental in promoting the system and rolling it out. National implementation started in high prevalence areas, which allowed the system to be tested more thoroughly in the final stages of Public Beta. The key challenges are presented by the variation in local pathways and processes, different levels of engagement from clinicians and the need to ensure the system is embedded in low prevalence areas.

To ensure service level agreements are met with the supplier in the future, a user group, regular service review and plans to identify and fix bugs and prioritise change request are in place. Key performance indicators will measure performance of helpdesk support, system availability and functionality. Reports will measure local and national performance against standards and the data quality report will support Specialist Haemoglobinopathy Teams ensure that all records are complete, and all babies are accounted for [9].

## Figures and Tables

**Table 1:** User group.

Laboratory scientists/directors	5
Haematologists/paediatricians	5
SCT specialist nurse/counsellors	2
Patients societies/parent representatives	4
PHE information & governance leads/programme manager	3
NHR CEO	1
Newborn outcomes team at KCL administrator/analyst/paediatrician	3

**Table 2:** Key findings, pain points and recommendations from Discovery.

Findings	Recommendations
There is currently no consolidated list of patients that people have submitted data on and are under their care, which means that relevant individuals have no visibility of the patient’s care beyond their step in the pathway. Without this access they rely on other departments for this information, and the process of requesting this data can be manual and time consuming.	A future solution should allow people who submit data to the programme to be able to view and export a consolidated list of all relevant patients.
There is duplication of effort inputting and submitting data. With no integration between systems, users are required to input the same data multiple times. For example, when haematologists receive a new patient, commonly they will need to input the same information on the NHR.	A future solution needs to, where possible, reduce the amount of duplicated effort. Thus, how a future solution could interact with existing systems to avoid the need to enter data more than once needs to be explored.
There is a lack of feedback from the newborn outcomes team when users have submitted the data. Many people interviewed felt they would benefit from a feedback mechanism and reports to improve their overall performance.	The future solution should include automatic confirmation when data has been received or uploaded. There should be functionality to allow reports to be created centrally. The ability for users outside of the central team to pull their own reports from a predefined reporting list should also be considered.
Poor quality data is submitted when users do not use the prescribed templates for submitting data, this requires a lot of manual effort to cleanse and reformat the data. It also makes it difficult to produce accurate reports if there is data missing.	There needs to be a clearly defined dataset. The future solution will need to have data validation rules to limit individuals from entering data that shouldn’t be on there and ensuring data quality and completeness.
The current process requires a lot of manual chasing of individuals for data. Eliciting information from haematologists/sickle cell counsellors can be challenging.	The future process will need to make sure that there are incentives to drive user adoption and ensure that data is submitted to the programme. For example, reducing their overall work effort. The new solution will also need to be user-friendly so that individuals are not put off submitting data.

**Table 3:** MoSCoW priority scale description.

Must	A key requirement, without which the system has no value
Should	An important requirement that must be delivered, but, where time is short, could be delayed for a future delivery. This should be a short-term delay.
Could	A requirement that would be beneficial to include if it does not cost too much or take too long to deliver, but that is not central to the project objectives.
Would	A requirement that will be needed in the future, but that is not required for this delivery.

**Table 4:** Examples of user stories.

“As a lab user I need diagnostic results so that I can QA the service and I need access to performance reports so that I can return data to PHE”
“As a paediatric haematologist I need to be able to pull data from the NBO system onto the NHR when parents’ consent so that I can avoid duplication of effort”.
“As a specialist nurse I need to be able to refer babies into treatment services so that I can ensure care is provided at the right centre”.
“As any user I need to visualise the patient’s progress along the pathway so that I am assured of safe handover from screening into treatment services”
